# Dietary pine pollen induces masculinization in Nile tilapia (*Oreochromis niloticus*, L. 1758) by modulating sex-biased gene expression and steroid hormone profiles

**DOI:** 10.1186/s13293-026-00950-5

**Published:** 2026-07-08

**Authors:** Ivan Abaho, Clifford L. W. Jones, Peter Akoll, Charles Masembe

**Affiliations:** 1 Bulindi Zonal Agricultural Research and Development Institute, National Agricultural Research Organization, P.O. Box 101, Hoima, Uganda; 2 Department of Ichthyology and Fisheries Science, Rhodes University, P.O. Box 94, Grahamstown, South Africa; 3 Department of Zoology, Entomology and Fisheries Sciences, Makerere University, P. O. Box 7062, Kampala, Uganda

**Keywords:** Aquaculture, Phytoandrogens, Sex change, Sex differentiation, 17α-methyltestosterone

## Abstract

**Background:**

The health and environmental risks associated with using 17-methyltestosterone hormone (MT) to produce all-male tilapia stocks limit the sustainability of aquaculture. As such, plant-based products with the potential to induce masculinisation in tilapia are gaining considerable attention as potentially safe, nature-based alternatives. However, the underlying mechanism for sex change induction by plant products remains unknown. The present study used pine pollen (PP) to study the processes modulating female-to-male sex change in three-day-old all-female Nile tilapia (*Oreochromis niloticus*).

**Methods:**

Experimental fish were fed either a basal diet supplemented with 1,280 mg PP kg^− 1^ from 3 to 30 days post-hatch (dph), or the same basal diet incorporated with 60 mg MT kg^− 1^ (MT treatment), or the basal diet only (CT treatment. After 30 dph, fish in all treatments were fed only a basal diet up to 120 dph.

**Results:**

Histological observations showed that female-to-male sex change occurred between 21 and 45 dph in MT and PP treatments. During this period, gonadal tissue in PP-treated fish progressively shifted from undifferentiated germ cells toward testes dominated by spermatogonia and spermatocytes. In contrast, MT treatment accelerated spermatogenic progression, with gonads exhibiting advanced germ cells, including spermatids and spermatozoa, reflecting complete masculinisation. Conversely, fish in the CT group underwent normal ovarian differentiation between 21 and 30 dph, characterised by ovarian cavity formation and oocyte-like germ cells. These gonadal histological changes were closely related to temporal changes in sex-biased gene expression and steroid hormone profiles. The PP and MT treatments significantly up-regulated male sex gene expression (*dmrt1* and *amh*) and down-regulated female transcripts (*cyp19a1a* and *foxl2)*, whereas an opposite pattern was observed in the CT group. Correspondingly, sex steroid concentrations (testosterone and 11-ketotestosterone) were also significantly elevated in both PP- and MT-treated fish, consistent with higher proportions of male individuals (PP: 77.8 ± 2.9% and MT: 97.8 ± 1.1%), unlike the CT group, where fish remained all-female with significantly higher 17β-estradiol levels.

**Conclusion:**

These findings imply that PP induces female-to-male sex change in Nile tilapia by disrupting the expression of sex-biased genes and, consequently, the androgen-to-estrogen balance. However, further studies are required to enhance the androgenic potency of PP.

## Abstract (plain language summary)

Nile tilapia mature early and spawn prolifically, leading to overcrowding in aquaculture systems, which causes resource competition and stress, resulting in stunted growth. All-male tilapia stocks are preferred as they grow faster and larger than females. However, producing all-male tilapia typically involves the synthetic chemical 17α-methyltestosterone (MT), which poses health and environmental risks. Recent studies suggest that plant-based alternatives, such as pine pollen (PP), could safely and affordably masculinize tilapia. This study tested PP as a natural alternative to MT by feeding all-female tilapia three diets: one with PP, one with MT, and one control (no PP or MT). The fish were fed these diets from 3 to 30 days old, then switched to a control diet until 120 days. Results showed that both PP and MT successfully converted females into males between 21 and 45 days, whereas the control group remained all-female. This masculinization was linked to the increased expression of male sex genes and the reduced expression of female sex genes in both the PP and MT groups. Consequently, male sex steroids were elevated, promoting female to male sex change. Up to 78% of the PP group and 98% of the MT group became males, while all fish in the control group remained females. The study suggests PP could be a natural method for sex control in tilapia farming.

## Highlights

All-male Nile tilapia grow faster and larger than females, offering potential aquaculture benefits.

Pine pollen acts as a natural alternative to 17-methyltestosterone, inducing female-to-male sex change in Nile tilapia.

Dietary pine pollen shifts sex ratios, resulting in a predominantly all-male population, by modulating sex-biased gene expression and steroid hormone profiles

Pine pollen offers a promising, safer, and nature-based approach for controlling unwanted breeding in Nile tilapia aquaculture.

## Introduction

Nile tilapia (*Oreochromis niloticus*) is a gonochoristic teleost that exhibits an XX/XY sex-determining system [1, 2]. During early sex development, the gonadal primordium is bi-potential and hence can develop into either a testis or an ovary. The sex differentiation process, however, is controlled by a combined effect of sex-specific genes and sex steroids [3–8]. Among the sex-specific genes, the Y-linked duplicate of the anti-müllerian hormone (*amhy*) is the master sex-determining gene that activates the sex differentiation network, thereby regulating the endocrine environment and consequently directing the sexual fate of the bi-potential gonadal primordium [5, 9–11]. Among the genes involved in female sex differentiation are cytochrome P450, family 19, subfamily A, polypeptide 1a (*cyp19a1a –* an aromatase gene); forkhead box L2 (*foxl2*), and factor in the germline alpha (*figla*). Doublesex and mab-3 related transcription factor 1 (*dmrt1*); gonadal soma-derived factor (*gsdf*); SRY-box transcription factor 9 (*sox9*); and anti-müllerian hormone (*amh*) are responsible for testicular differentiation [3, 5, 12–17]. The male and female-promoting networks act antagonistically through gene up- or down-regulation, to re-program the sex differentiation process [18–22]. For instance, higher expression of *dmrt1* and *amh* represses *foxl2* and *cyp19a1a* to trigger testicular differentiation [18, 19, 23–29]. Conversely, the suppression of *dmrt1* and *amh* results in upregulation of *foxl2*, which activates the expression of *cyp19a1a* to induce ovarian development [18, 23, 26, 27, 30–34].

The antagonistic behaviour of sex-related genes during sex differentiation regulates the estrogen and androgen synthesis cascade in Nile tilapia [18]. Among the estrogens, 17β-estradiol (E2) is vital in the differentiation and maintenance of the ovaries of the fish, while 11-ketotestosterone (11-KT) plays a similar role for the testes [10, 20, 35–39]. Notably, testosterone (T), an androgen, acts as a precursor of 11-KT and E2 [31, 40, 41]. A shift in the expression of the sex-biased genes alters the bioconversion rates of T to either 11-KT or E2, disrupting the androgen-to-estrogen hormonal balance and subsequently directing the gonadal differentiation process [18, 42–44]. Higher expression of the aromatase gene (*cyp19a1a*) catalyses the conversion of T to E2, hence overriding testicular differentiation, while promoting female sex development [31, 33, 40, 41, 45]. In contrast, aromatase inhibition averts the aromatisation of T to E2, while enhancing the conversion of T to 11-KT, thereby depleting estrogen levels in the differentiating gonad to elicit male sex development [6, 31, 37, 46–50].

However, the natural gonadal differentiation network can be overridden by extrinsic factors, mainly hormones and temperature, irrespective of the genetically determined sex [20, 33, 51–58]. For example, androgens, mainly 17α-methyltestosterone (MT), inhibit estrogen synthesis, subsequently inducing female-to-male sex inversion [49, 52, 59–62]. Exposing Nile tilapia fish to MT, prior to gonadal differentiation, results in stimulation and repression of the male and female sex genes, respectively, and eventually induces masculinisation [6, 54, 63, 64]. Exogenous androgen treatment subsequently enhances endogenous androgens in the fish, mainly 11-KT, while repressing estrogen production, particularly E2, ultimately inducing primordial germ cells to differentiate in a testicular direction, whereas female phenotypic sex development is suppressed [49, 52, 65–68]. Commercial Nile tilapia hatcheries have harnessed this phenomenon to produce all-male stock that exhibits superior growth compared to females, and hence is commercially preferred. This is primarily achieved using 17α-methyltestosterone (MT), with 60 mg MT kg⁻¹ diet identified as the most effective dose [69–72].

The use of MT is associated with potential hazardous effects on humans and biodiversity, which are increasingly becoming a public concern [73–76]. Prolonged exposure to MT poses a health risk to hatchery personnel [77], including hepatotoxicity and fetotoxicity [78–80], menstrual irregularities and breast atrophy in women, as well as impotence and prostatic hypertrophy in men [81]. In the environment, MT and its metabolites have the potential to disrupt the endocrine and reproductive systems of non-target aquatic organisms [76, 82–84]. As such, bioactive compounds from plant extracts are proposed as a safe, eco-friendly alternative for sex control in tilapia. Previous studies demonstrated that pine pollen (PP) has the capacity to reverse sex in Nile tilapia [62, 70, 85–88]. Nonetheless, the mechanism underlying the PP-induced female-to-male sex change is unknown. To understand the role of PP in sex masculinisation, the present study examined the temporal changes in the expression profiles of sex-biased genes: *cyp19a1a*, *foxl2*, *amh*, and *dmrt1*, using sexually undifferentiated all-female (XX) Nile tilapia. Additionally, changes in the levels of three primary sex steroids: T, 11-KT, and E2, and the associated histological changes in the gonads were investigated.

## Materials and methods

### Experimental fish production and rearing

Neomale individuals (XX males) were obtained by feeding sexually undifferentiated Nile tilapia diets containing PP and were identified using a Y-linked duplicate of *amh* [9]. Genomic PCR amplification of the Y-specific fragment was performed to screen for *amhy*-positive (XY or YY) fish only. The samples without *amhy* amplification (amhy-) were denoted XX and used as neo-males, i.e., spermiating genetic female individuals. The identified XX males were crossed with normal Nile tilapia females to produce all XX progeny. Matings were performed at a ratio of three females to one neo-male in 60 m^3^ concrete circular tanks under natural photoperiod and water temperature (27–30 °C). After 14 days, eggs were collected from the mouths of the females and incubated at 27 ± 1.0 °C in Zuger jars until hatching. All-female fish (3 days post hatch - dph and mean body weight mass: 0.025 ± 0.002 g) were collected randomly and stocked into nine continuously aerated experimental plastic tanks (each filled with 120 L of water) at 300 fish per tank. The tanks were operated as a flow-through system, with a flow rate of 1.2 L min^− 1^. To maintain water quality within the optimal range for Nile tilapia growth, culture water was monitored and regulated daily using a digital water quality meter (In-Situ SmarTROLL™ MP, USA). Water temperature and dissolved oxygen were maintained at 27 ± 0.8 °C using thermostatic heating rods (Sera Aquarium heater thermostat, Germany) and 5.5 ± 0.20 mg L^− 1^ by continuous aeration, respectively, and pH at 7.2 ± 0.30. Ammonia-nitrogen was monitored daily using a commercial freshwater aquaculture kit (LaMotte Company Ltd, USA) and kept below 0.2 mg L^− 1^. All uneaten food and other debris were removed from the culture unit daily, in the morning hours before feeding commenced.

### Pine pollen (PP) and 17α-methyltestosterone (MT) hormone treatment

**Fig. 1:**
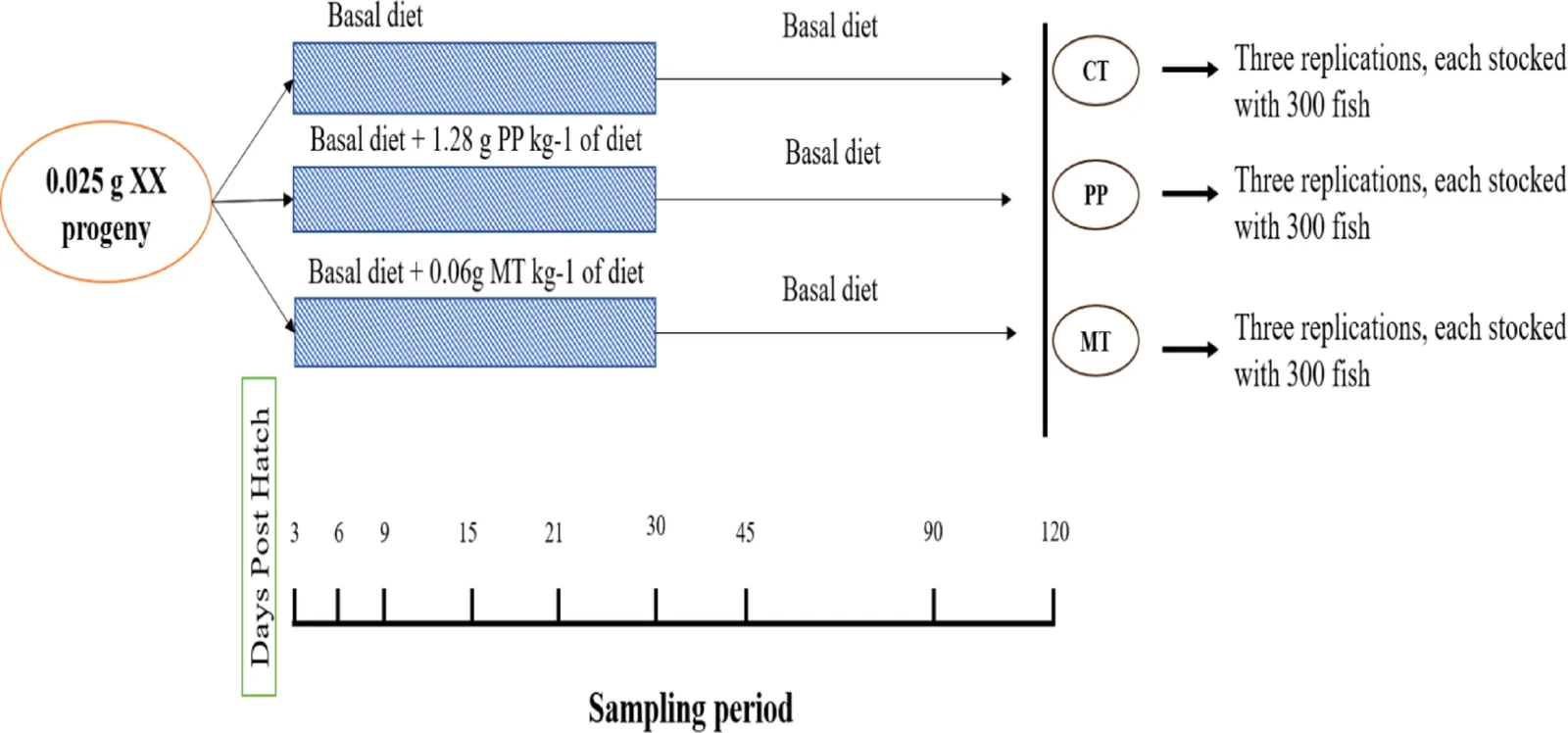
Schematic diagram of the experimental design illustrating the applied three treatments: CT (Control treatment; negative control), PP (pine pollen treatment; 1,280 mg PP kg^− 1^ of diet), and MT (17α-methyl testosterone hormone; 60 mg MT kg^− 1^ of diet)

The present study used the same basal diet and a commercially available pine pollen (PP; Jiangsu Qinshantang Health Industry Co., Ltd, Nanjing, China) harvested from *Pinus tabulaeformis* species, and 17α-methyltestosterone hormone (purity ≥ 98%) (MT; Sigma Chemical Co., St Louis, MO, USA), as used in the previous study [70]. The optimal PP dose determined in that study (1,280 mg PP kg-1 feed) was adopted in the present experiment, and diet preparation and storage followed procedures described therein. Fish were fed a control basal diet with no PP treatment (negative control), or the same basal diet treated with 1,280 mg PP kg^− 1^ of diet, or the same basal diet treated with 60 mg MT kg^− 1^ of diet and with no pine pollen treatment (positive control). Each dietary treatment was fed to the fish from 3 to 30 dph (Fig. 1). To confirm whether the sex change was complete, fish in all treatment groups were fed only a basal diet for an additional 84 days from 31 to 120 dph. Throughout the experiment, the fish were fed to apparent satiation six times (from 3 to 30 dph) and four times (from 31 to 120 dph) daily during daylight hours.

### Tissue sampling

At different stages of the experiment (Fig. 1), a random sample of fish was picked from the experimental tanks and euthanised with 250 mg L^− 1^ of MS-222 (Sigma-Aldrich) for gonadal extraction. At each sampling point, 9 samples, each for steroid analysis and histological examination, were collected (*n* = 18 per treatment, i.e., 6 samples per replicate). At the same time, gonadal tissues from two fish per replicate (*n* = 6 per treatment) were sampled for gene expression analysis.

Between 3 and 21 dph, the fish were too small to accurately excise the gonad. As such, the non-gonadal tissues, i.e., heads, tails, and most visceral organs, were carefully removed using sterile surgical blades, retaining only the central trunk region containing the gonadal primordia. This was done to enrich the tissue sample for gonadal cells, minimize contamination from surrounding non-target tissues, and improve the accuracy and sensitivity of subsequent molecular and hormonal analyses. From 30 to 120 dph, the gonads were dissected using fine forceps. During gonadal extraction, RNALater (Sigma-Aldrich, St. Louis, USA) was dropped onto the coelomic epithelium to stabilise the RNA. Both the trunks and gonads were immediately placed in 1.5 mL tubes containing RNALater to preserve RNA integrity. The samples were kept at -20 °C until RNA extraction. The samples for steroid analysis were kept at -30 °C until use, while gonadal histological samples were immersed in Bouin’s solution and stored at room temperature. For sex-ratio examination, 90 fish were sampled from each treatment at 120 dph, their gonads were extracted and preserved in Bouin solution until histological analysis for phenotypic sex examination.

### Gonadal histology and sex ratio examination

The Bouin-fixed samples were loaded into tissue cassettes and immersed in two changes of 50% ethanol to remove the fixative solution. The samples were then dehydrated by immersion in an ascending ethanol series with concentrations of 70%, 80%, 90%, and 95%, and finally in three changes of absolute ethanol, each for one hour. The dehydrated samples were cleared in three changes of xylene at an interval of one hour each and, thereafter, impregnated with two changes of molten paraffin wax. Sample dehydration, clearing, and infiltration were all done using an automated benchtop tissue processor (LEICA TP1020, Germany). The impregnated tissues were cross-sectioned at 4 μm using a rotary microtome (LEICA RM2235, Germany). The obtained histological sections were stained with haematoxylin-eosin (H & E) and finally examined and classified as ovary, testis, or ovotestis under a light compound microscope (Nikon Eclipse c*i*, China). Photomicrographs were taken using a digital camera (Nikon Digital Sight, Japan) mounted onto a microscope. The developmental stages of the gonad tissues were classified based on Bancroft’s histological criteria [89]. At 120 dph, the sex ratio in each treated group was determined following the histological analysis results, and percentage masculinisation was calculated based on the number of males in each treatment group.

### Sex steroid hormone extraction and assays

Freshly stored (-30 °C) trunks and gonads were homogenised in a 1.5 mL tube using a pestle, one at a time to prevent cross-contamination. To each homogenised sample, 0.5 mL of double-distilled water was added and thoroughly mixed. The organic solvent extraction technique was then utilised for all the steroids [90]. The extracted hormones were dissolved in 500 µL of the ELISA buffer for T and 11-KT, while for E2, reconstitution was done with 300 µL of the buffer (Cayman Chemical Co., USA). The extracted steroids were stored at -80 °C until the assay was done. The concentrations of T, 11-KT, and E2 were determined using enzyme-linked immunosorbent assay (ELISA) kits (T: 582701, 11-KT: 582751, and E2: 501890), following the manufacturer’s instructions (Cayman Chemical Co., USA). For each steroid, one assay was done, and the mean intra-assay coefficients of variation were 6.12 ± 1.54%, 0.25 ± 1.25%, nd 7.20 ± 1.43% fr T, 11-KT, and E2, respectively.

### Gene expression analysis

#### RNA extraction and cDNA synthesis

Total RNA was extracted using the Quick-RNA™ MiniPrep Plus Kit (Zymo Research, Tustin, CA, USA) according to the manufacturer’s protocol. The concentration and purity of the RNA were measured on a NanoDrop spectrophotometer (NanoDrop 2000c, Thermo Scientific, Massachusetts, USA) at absorbance wavelength ratios of 260/280 and 260/230, respectively. The integrity of RNA was assessed by electrophoresis on a 2% agarose gel stained with ethidium bromide, along with a GeneRuler 1 kb Plus DNA ladder (ThermoFisher Scientific, USA). Thereafter, 2 µL of total RNA from each sample was reverse transcribed to cDNA using ProtoScript II First Strand cDNA synthesis kit (New England Biolabs, USA), following the manufacturer’s instructions. The obtained cDNA was stored at -20 °C until use.

#### Quantitative real-time polymerase chain reaction (qPCR)

The cDNA was subjected to qPCR analysis using a 96-well QuantStudio™ 7 Pro (A43055) real-time PCR system (Applied Biosystems, Carlsbad, CA, USA). Gene-specific primers for *cyp19a1a* and *foxl2* [91], *amh* [51, 92], and primers for *dmrt1* [93] were used. Beta-actin (*β-actin*) was used as an internal control [94–96]. The nucleotide sequences of the primers for each gene are presented in Table 1 below. The samples were analysed in duplicate, with reactions performed using Luna Universal qPCR Master Mix (New England Biolabs, USA) according to the manufacturer’s instructions. Each reaction volume was 20 µL, comprising 2 µL of cDNA, 7 µL of nuclease-free water, 0.5 µL (10 pmol) of each forward and reverse primer, and 10 µL of Luna Universal qPCR Mix. A non-template control, with no cDNA, was also included in each qPCR. The PCR reaction conditions were as follows: an initial denaturation at 95 °C for 30 s, 40 cycles of 95 °C for 5 s, 60 °C for 30 s, and 72 °C for 30 s. To confirm whether the qPCR amplified the intended target, the products were electrophoresed on a 2% agarose gel (125 V, 30 mn), stained with ethidium bromide, and run alongside a 50 bp DNA size marker (Bioline, United Kingdom). A single clear band of the desired size was obtained for all genes. The expression of all genes was measured from cDNA derived from the same fish per treatment, allowing comparisons among genes within each treatment. Relative quantification of each target gene was determined following Pfaffl mathematical model [97]. The data were normalised using *β-actin* as the reference gene.

**Table 1:** Primers and related information of the sex-biased genes used in the study

Gene	Primer Name	Primer sequence (5’ to 3’)	Size product (bp)	Tm	Accession number
*amh*	amh-Forward	AAGCAGCGCAAACATTAACA	169	53.2 °C	EF512167*
	amh-Reverse	GTTCCAGTCCACAACCTCCA		59.4 °C	
*cyp19a1a*	cyp19a1a- Forward	AGGCGATGAGTCCTGTAGGCTTAG	346	64.4 °C	AF472620*
	cyp19a1a-Reverse	TTATTGTAGTAGTTGCTGGCTGTGC		61.3 °C	
*dmrt1*	dmrt1-Forward	TGAGCCAGGACAAACAGAGT	210	59.9 °C	AF203489.1*
	dmrt1-Reverse	CCATGACTCTCTGCCTCTCC		59.3 °C	
*foxl2*	foxl2-Forward	TGGCAGAACAGCATCAGACACAAC	313	62.7 °C	AY554172*
	foxl2-Reverse	TGTAGGACATCGGAGTGGGTGGCT		66.1 °C	
*β-actin*	*β*-actin-Forward	GGCATCACACCTTCTACAACGA	332	60.3 °C	XM_003443127^**†**^
	*β*-actin-Reverse	ACGCTCTGTCAGGATCTTCA		57.3 °C	

*amh*: anti-müllerian hormone hormone; c*yp19a1a*: cytochrome P450, family 19, subfamily A, polypeptide 1a; *dmrt1*: doublesex and mab-3 related transcription factor 1; *foxl2*: forkhead box protein L2; *β-actin*: beta-actin; bp: base pair; Tm: melting temperature; *: GenBank accession number; ^**†**^: RefSeq accession number

### Data analysis

The data were expressed as mean ± standard error (SE) for each treatment. The statistical differences in relative gene expression and sex steroid levels across treatments over the experimental period (days post-hatch) were analysed using a repeated-measures analysis of variance (RM-ANOVA). When significant interaction between the main effects was obtained, Tukey’s HSD post-hoc test was used to compare treatment means at all times. Simple linear regression analysis was also performed to test for an increasing or decreasing trend in gene expression patterns and in sex steroid levels during the experimental period. The differences in masculinisation among treatments were examined using a one-way analysis of variance (one-way ANOVA). The differences between means or interactions between main effects were considered statistically significant at *P* < 0.05. All statistical analyses were performed with Statistica^®^ statistical software package (TIBCO Software, Palo Alto, USA, version 14.0.1). The histograms were all generated using Origin 2022b software (OriginLab Corporation, USA).

## Results

### Histological structure of gonads

**Fig. 2:**
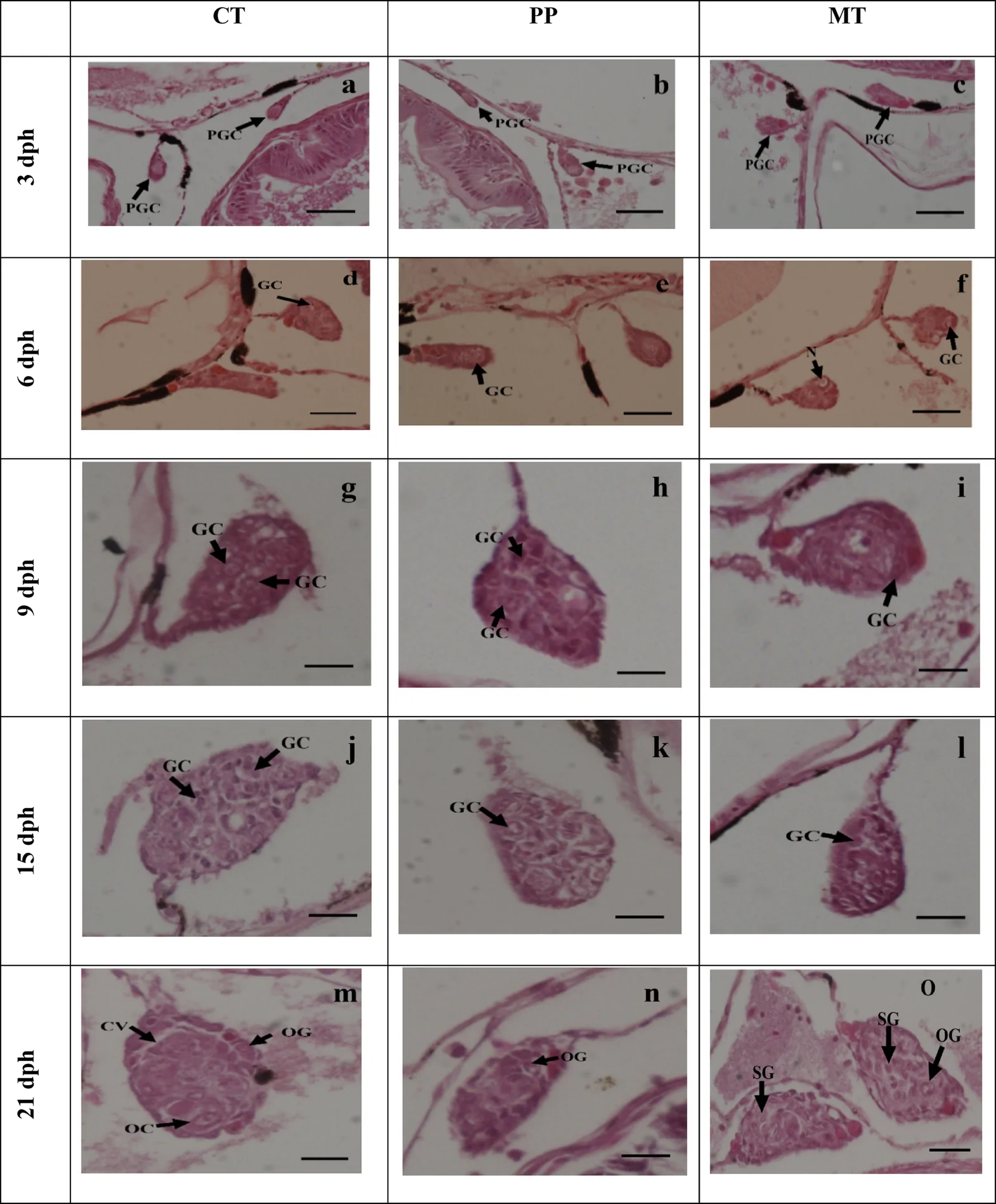

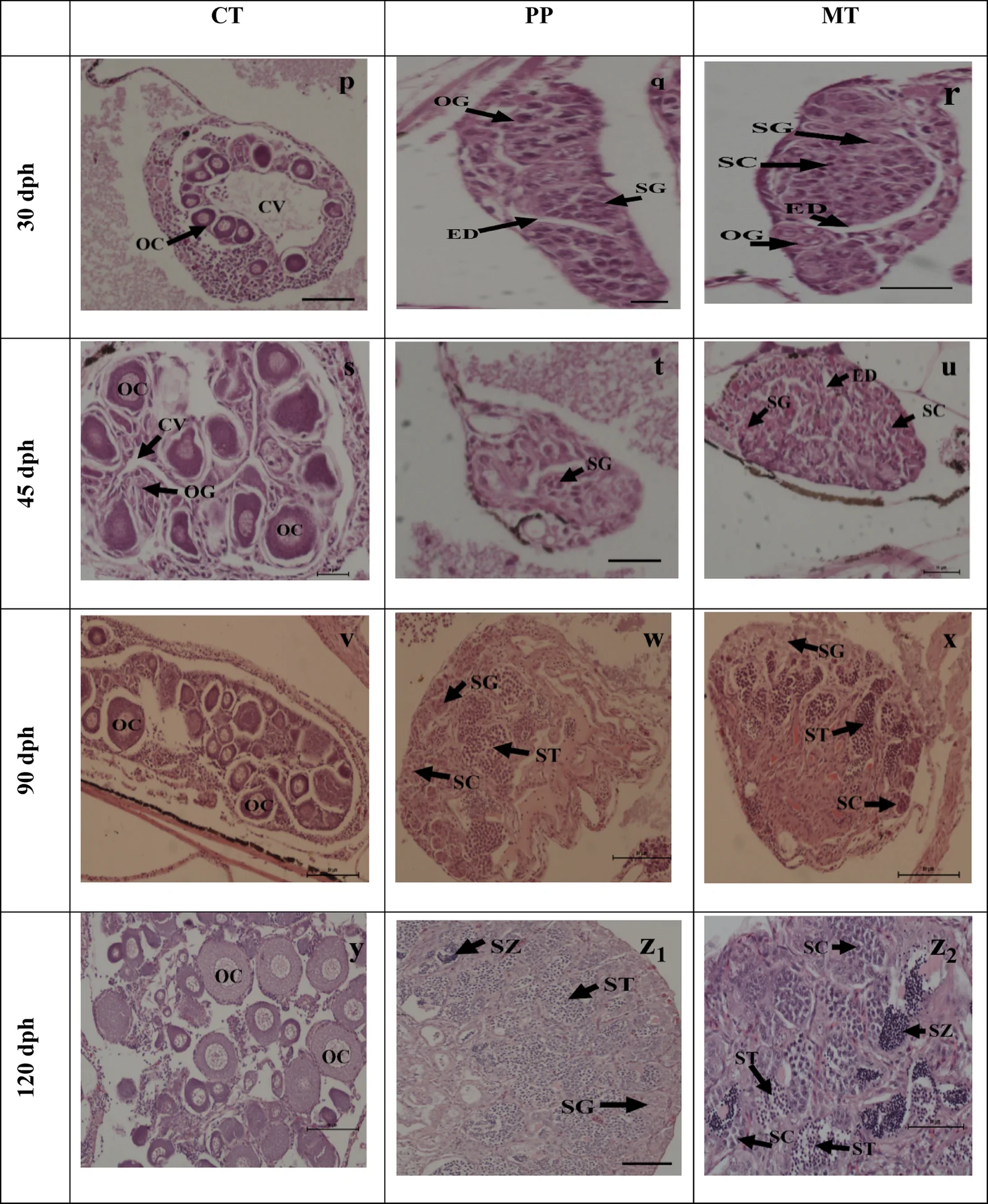
Changes in the gonadal histology of all-female Nile tilapia from 3 to 120 days post-hatch (dph). CT: fish fed only on basal diet from 3 to 120 dph; PP: fish fed a basal diet supplemented with 1,280 mg pine pollen (PP) from 3 to 30 dph, and after that, a basal diet from 31 to 120 dph; MT: fish fed a basal diet supplemented with 60 mg of 17α-methyltestosterone (MT) for 28 days from 3 dph, and thereafter a basal diet up to 120 dph. PGC: primordial germ cell; GC: germ cell; N: nucleolus; CV: ovarian cavity; OG: oogonia; OC: oocyte; ED: efferent duct; SG: spermatogonia; SC: spermatocyte; ST: spermatid; SZ: spermatozoa. Scale bar: a - u = 10 μm and v-z_2_ = 50 μm

Gonadal tissues of individuals at 3 dph, prior to exposure to PP and MT, appeared as elongated thin filaments in the abdominal cavity, consisting of primordial germ cells with no signs of directional differentiation (Figs. 2a, b, and c). At 6 dph, the paired arrangement of the gonads became more distinct, suspended into the coelomic cavity, extending caudally from the pectoral fins (Figs. 2d, e, and f), but exhibited no pronounced variations in histological features across the treatments. At 9 dph, the gonads were appreciably enlarged, corresponding to the increasing germ cell numbers as active germ cell proliferation began (Figs. 2g, h, and i). The resultant cells, however, were relatively smaller than the primordial germ cells observed at 3 and 6 dph. Furthermore, the number of germ cells present in CT- and PP-treated individuals (CT: Fig. 2g and PP: Fig. 2h) was higher than in the gonadal tissues obtained from the MT-treated fish (Fig. 2i). At 15 dph, the gonadal size and the corresponding number of germ cells were evident and higher than in the preceding stages. Further, the rate of increase of germ cells slowed in PP and MT-treated fish, and thus fewer germ cells were observed than in the CT group (Figs. 2j, k, and l). At 21 dph, the first signs of ovarian differentiation were observed in the CT treatment, with the initiation of ovarian cavity formation, which appears to begin from the anterior region towards the caudal portion of the gonads (Fig. 2m). At this stage, the gonads contained small, rounded oogonia with a spherical nucleus occupying the entire cell. Meanwhile, in the MT-treated fish, the female-to-male sex change process commenced with gonadal differentiation from ovarian to testicular (Fig. 2o). Although some ovarian tissue remained in the gonads, spermatogonial formation was initiated. In the PP-treated fish, the gonads were still developing as ovaries by 21 dph, having only oogonial tissues, but with smaller gonads and fewer germ cells (Fig. 2n) compared with CT-treated individuals. At 30 dph, the structure of the ovary in the CT-treated fish was very distinguishable with a perfect construction of the ovarian cavity, showing normal ovarian differentiation and an increasing number of oocytes (Fig. 2p). In the MT gonadal tissues, female-to-male sex differentiation was more prominent with lobular testicular tissues, high proliferation of spermatocytes with few oocytes, present in the gonads (Fig. 2r), and a more distinct efferent duct compared to 21 dph. In the PP-treated fish, the female-to-male sex differentiation had also commenced, with the formation of the slit-like space, indicative of the forming efferent duct (Fig. 2q). The gonads were still ovotestes, i.e., contained both oocytes and spermatogonia.

At 45 dph, the CT-treated fish had a large ovarian volume, and the gonads were comprised of only fully developed oocytes (Fig. 2s). The gonads of PP-and MT-treated fish had sex inversed from females to males. In the PP treatment, the gonads consisted mainly of spermatogonia (Fig. 2t), whereas the MT group had gonads containing both spermatogonia and spermatocytes (Fig. 2u). The appearance of testicular tissues followed the reduction in the germ cells in PP and MT treatments. By 90 dph, sex differences were evident among the treatment groups. In the CT treatment, all fish possessed female gonadal tissues (Fig. 2v). The gonads from a significant proportion of the MT-treated fish were fully transformed into the testis, with definite testicular tissues at different stages of spermatogenesis, i.e., spermatogonia, spermatocytes, and spermatids (Fig. 2x). Likewise, most of the fish from the PP treatment exhibited male attributes (Fig. 2w). At the end of the experiment (120 dph), (a) CT group contained only female fish with normal mature ovaries containing oocytes (Fig. 2y), (b) MT-treated fish were all males with typical testes (Fig. 2z_2_), and (c) PP group consisted of eight male individuals (Fig. 2z_1_) and one female, with ovarian tissues similar to samples from the CT treatment. The level of maturity varied between the PP and MT-treated fish. The testicular lobules of the PP-treated fish were dominated by spermatogonia and spermatids (Fig. 2z_1_), while males from the MT treatment had gonads composed mainly of spermatids and spermatozoa (Fig. 2z_2_).

### Level of masculinisation of the experimental fish

The gonadal phenotypes of fish from each treatment were histologically determined at 120 dph. All the fish from the CT treatment remained females, while female-to-male sex inversion was observed in PP and MT-treated fish (Table 2). Overall, MT significantly induced female-to-male sex inversion (97.8 ± 1.1% males) compared with the 77.8 ± 2.9% males observed in the PP-treated group (ANOVA; F _(1, 4)_ = 40.5, *P* = 0.003). Notably, 5.6 ± 1.1% of the individuals in the PP-treated group had ovotestis, comprising mainly of spermatocytes and a few oocytes, implying only 15.6 ± 1.9% remained un-masculinised (XX-females).

**Table 2:** Gonadal phenotype and percentage masculinisation of all-female (XX) Nile tilapia fed a basal diet only (CT), or the same basal diet + 1,280 mg of PP kg-1 of feed (PP), or the same basal diet + 60 mg of MT kg-1 of feed (MT)

Treatment	Number of fish examined	Number of ovaries	Number of testes	Number of ovo-testes	Masculinization rate (% ± S.E.)
CT	90	90	0	0	0.0 ± 0.0
PP	90	15	70	5	77.8 ± 2.9
MT	90	2	88	0	97.8 ± 1.1

### Changes in 17β-estradiol levels of Nile tilapia among treatments

**Fig. 3:**
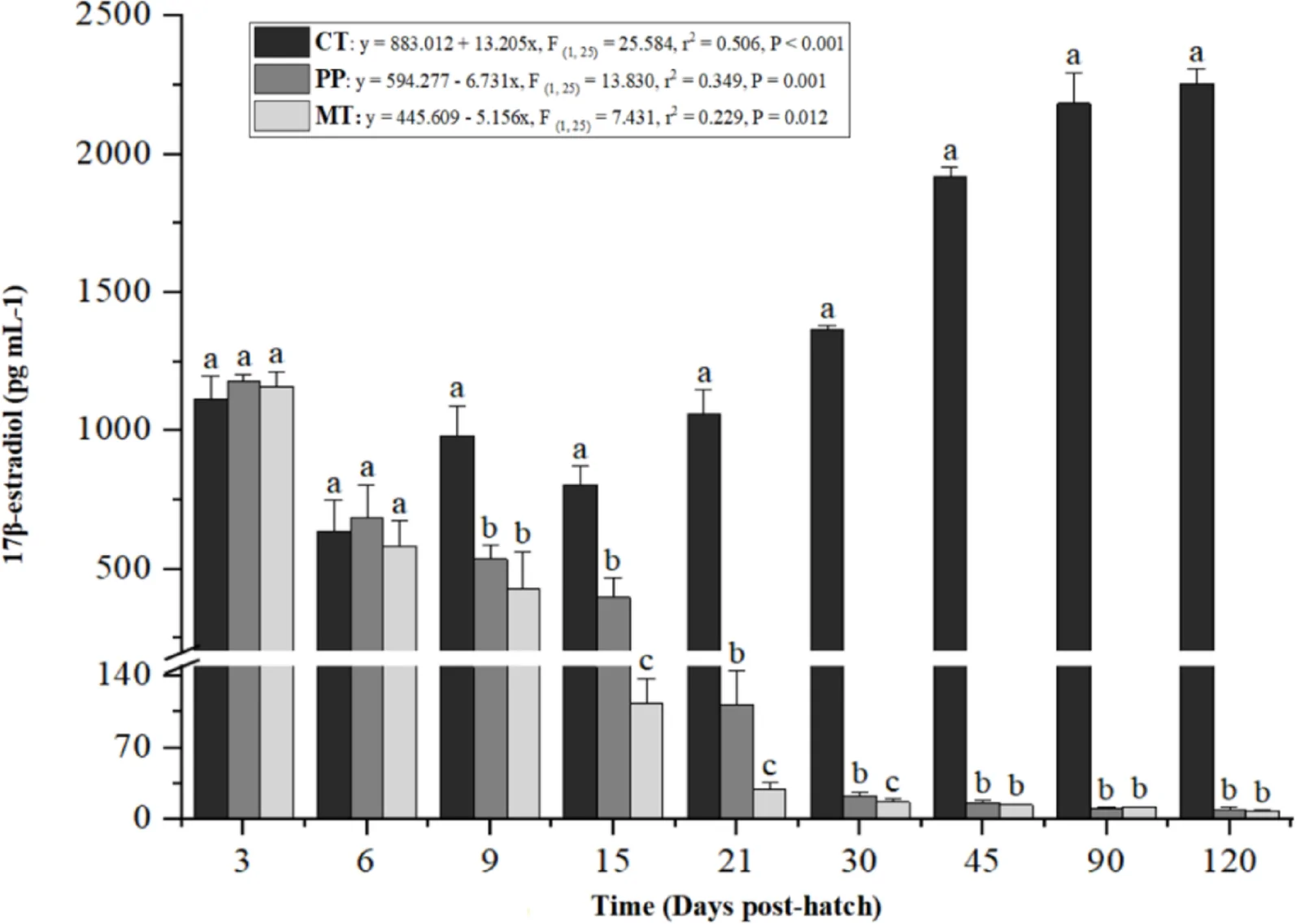
The changes in 17β-estradiol (E2) concentration (pg mL^-1^) in gonadal tissues of Nile tilapia fed only a basal diet (CT), or the same basal diet supplemented with 1,280 mg pine pollen kg^-1^ diet (PP), or the same basal diet supplemented with 60 mg 17α-methyltestosterone hormone kg^-1^ diet (MT). Data were expressed as the mean ± standard deviation (SE) for the nine fish samples from each treatment per sampling point. Different superscript letters above error bars indicate significant differences in the steroid concentration among treatments in the same time interval (Tukey’s HSD post-hoc, *P* < 0.05). The regression model equations present the changes in E2 concentration as a function of time for each treatment

The concentrations of 17β-estradiol (E2) in Nile tilapia among the treatments were significantly influenced by an interaction between fish rearing period and dietary treatment (RM-ANOVA; F _(16, 48)_ = 5.338, *P* < 0.001; Fig. 3). A significant negative relationship between E2 and time was observed in the fish from the PP (y = 594.277–6.731x, F _(1, 25) =_ 13.830, r^2^ = 0.349, *P* = 0.001; Fig. 3) and MT (y = 445.609–5.156x, F _(1, 25) =_ 7.431, r^2^ = 0.229, *P* = 0.012; Fig. 3) treatments.

The E2 levels were generally higher in the gonads of fish from the CT treatment, while consistently lower in PP and MT-treated fish (Fig. 3). From 3 to 6 dph, the steroid decreased in all treatments, and thereafter it significantly increased in the fish from the CT group until 120 dph (*P* < 0.05). However, E2 decreased continuously in the MT and PP treatments during the remaining experimental period, reaching the lowest levels at 120 dph (PP: 10.135 ± 2.168 pg mL^− 1^ and MT: 8.956 ± 0.247 pg mL^− 1^). From 15 to 30 dph, the steroid was significantly depressed in the MT treatment compared to the PP-treated fish (*P* < 0.05). However, E2 was also consistently suppressed in the PP-treated fish, reaching significantly similar levels as in MT treatment from 45 to 120 dph (*P* > 0.05).

### Concentration of 11-ketotestosterone in experimental fish

**Fig. 4:**
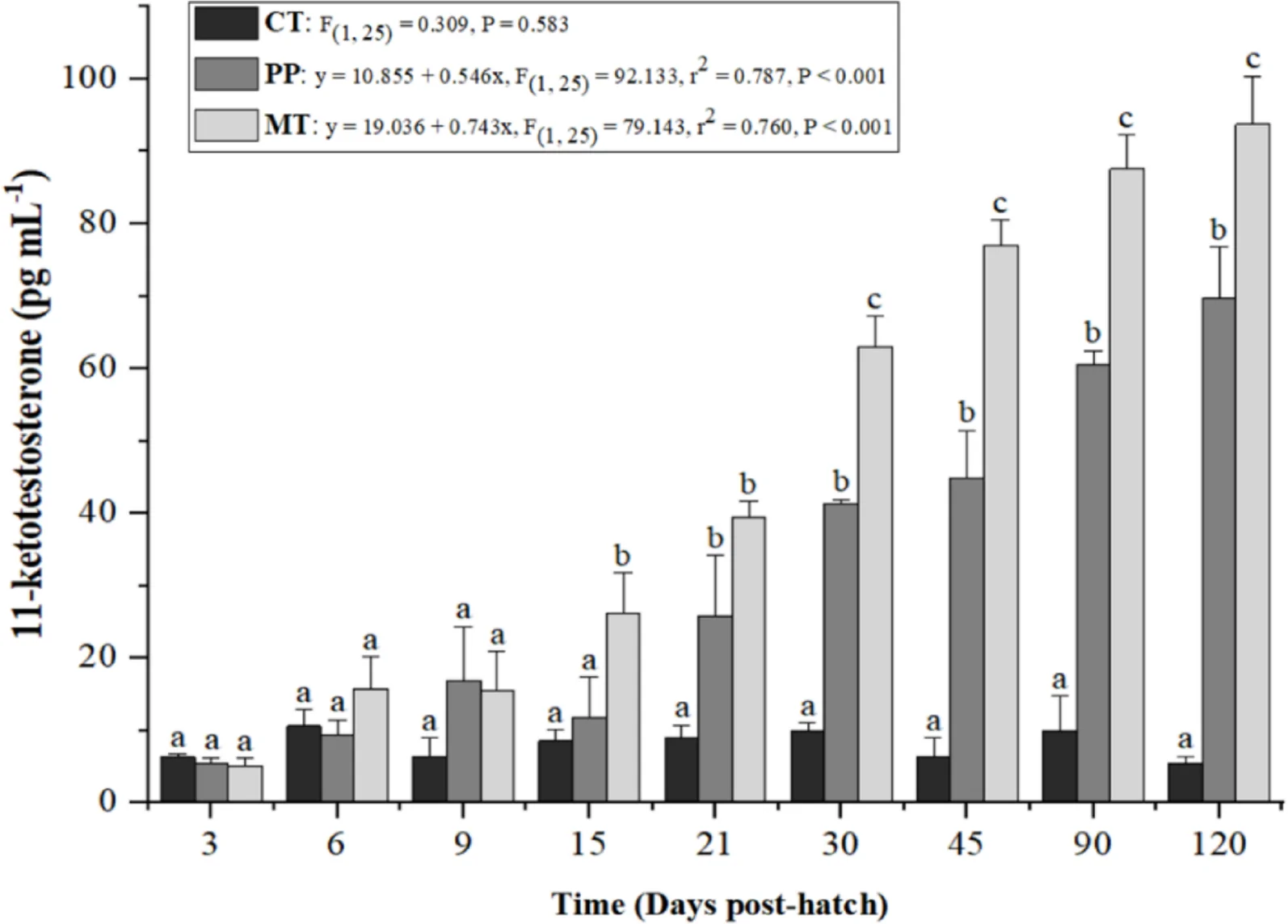
The 11-ketotestosterone (11-KT) concentration (pg mL^-1^) in the gonadal tissues of Nile tilapia fed only a basal diet (CT), or the same basal diet supplemented with 1,280 mg pine pollen kg^-1^ diet (PP), or the same basal diet supplemented with 60 mg 17α-methyltestosterone hormone kg^-1^ diet (MT). Data were expressed as the mean ± standard deviation (SE) of the nine sampled individuals for each treatment per sampling point. The significant differences in the steroid concentration among treatments are indicated by different superscripts above error bars in the same time interval (Tukey’s HSD post-hoc, *P* < 0.05). The regression model equations indicate the changes in 11-KT concentration as a function of time for each treatment

The concentrations of 11-ketotestosterone (11-KT) among the treatments were significantly influenced by an interaction between the fish rearing period and dietary treatment (RM-ANOVA; 11-KT: F _(16, 48)_ = 13.705, *P* < 0.001; Fig. 4). A significant positive relationship between the steroid concentration and time was observed in the PP (y = 10.855 + 0.546x, F _(1, 25)_ = 92.133, r^2^ = 0.787, *P* < 0.001; Fig. 4) and MT (y = 19.036 + 0.743x, F _(1, 25)_ = 79.143, r^2^ = 0.760, *P* < 0.001; Fig. 4) treatments up to 120 dph. However, no significant relationship between 11-KT and time was exhibited in the CT treatment (Regression analysis; F _(1, 25)_ = 0.309, *P* = 0.583; Fig. 4).

Higher levels of 11-KT were largely displayed in the PP and MT treatments than in the CT group throughout the experimental period. The steroid levels in the CT treatment were significantly low throughout the experimental period and did not exceed 10.62 ± 2.27 pg mL^− 1^. In contrast, 11-KT levels steadily increased in PP and MT-treated fish, although the increment was faster in the MT treatment. Maximum steroidal levels were obtained in the MT-treated fish (93.72 ± 6.67 pg mL^− 1^) at 120 dph compared to 69.64 ± 7.14 pg mL^− 1^ in the PP treatment. In the initial stages of the experiment, i.e., from 3 to 9 dph, no significant differences were observed in 11-KT levels among the treatments (*P* > 0.05). However, by 15 dph, the MT-treated fish had significantly higher steroid levels than the fish from the CT and PP treatments (*P* < 0.05). From 21 dph, a steady rise in 11-KT was observed in the PP treatment, and the steroid was significantly higher than in fish from the CT treatment until the end of the experiment (*P* < 0.05). A faster increase of 11-KT was also observed in the MT-treated fish, with the steroid becoming significantly higher than in the fish from the PP group from 30 dph till the end of the experiment (*P* < 0.05).

### Levels of Testosterone among treatment groups

**Fig. 5:**
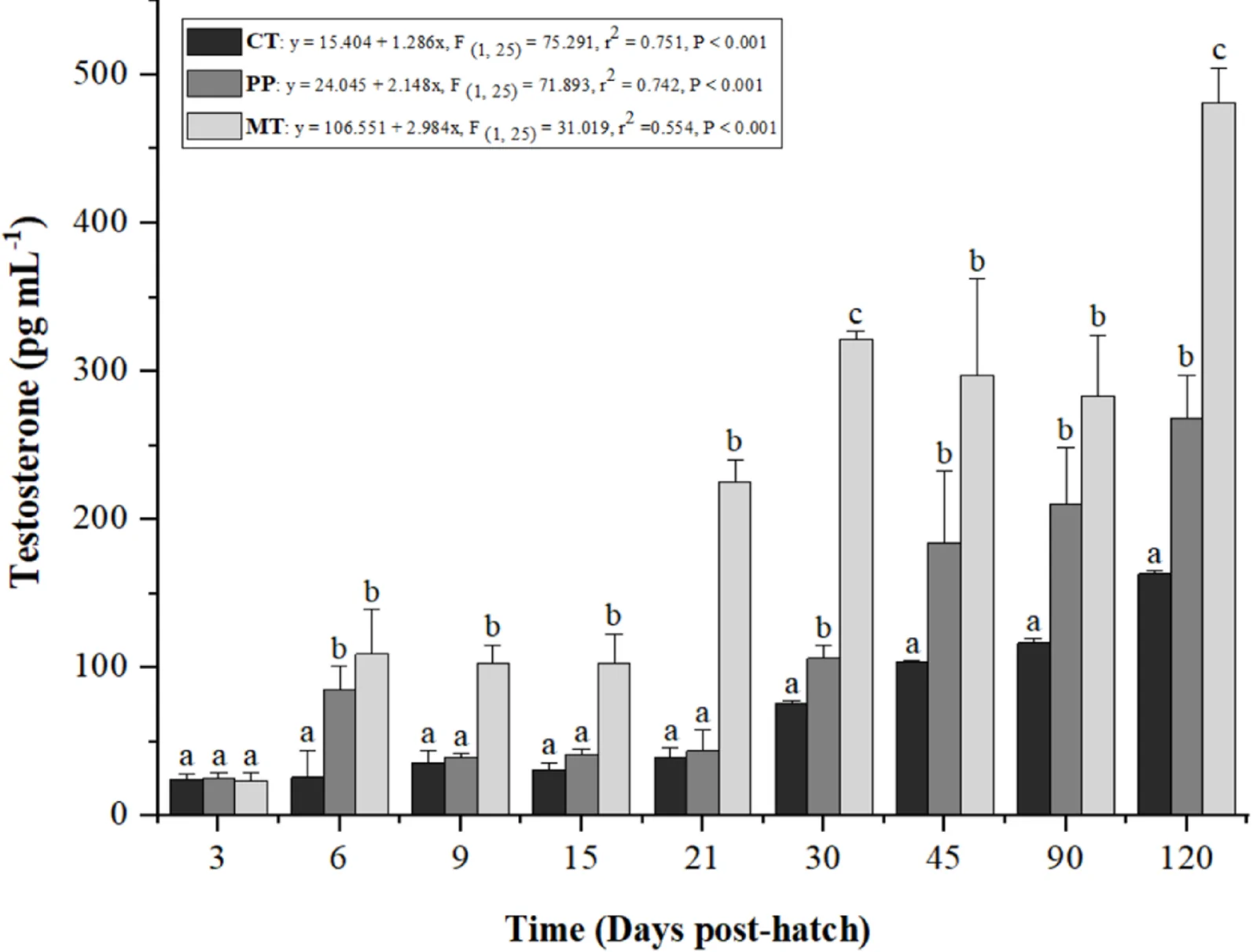
Testosterone concentration (pg mL^-1^) of Nile tilapia fed only a basal diet (CT), or the same basal diet supplemented with 1,280 mg pine pollen kg^-1^ diet (PP), or the same basal diet supplemented with 60 mg 17α-methyltestosterone hormone kg^-1^ diet (MT). All data were expressed as the mean ± standard deviation (SE) for nine randomly sampled fish individuals per treatment at each sampling point. Different superscript letters above error bars indicate significant differences in the steroid concentration among treatments in the same time interval (Tukey’s HSD post-hoc, *P* < 0.05). The regression model equations show the changes in the concentration of T over time for each treatment

The concentration of testosterone (T) in Nile tilapia among the treatments was influenced by a significant interaction between the fish rearing period and dietary treatment (RM-ANOVA; F _(16, 48)_ = 2.636, *P* = 0.005). In all the treatments, a significant positive relationship between the concentration of T and time was observed (CT: y = 15.404 + 1.286x, F _(1, 25)_ = 75.291, r^2^ = 0.751, *P* < 0.001; PP: y = 24.045 + 2.148x, F _(1, 25)_ = 71.893, r^2^ = 0.742, *P* < 0.001; and MT: y = 106.551 + 2.984x, F _(1, 25)_ = 31.019, r^2^ =0.554, *P* < 0.001; Fig. 5). Higher levels of the steroid were found in the MT and PP-treated fish than in the CT group, throughout the experimental period. At 3 dph, the levels of the steroid (T) did not significantly differ among the three treatments. Thereafter, T in the MT-treated fish increased and became significantly higher than in the CT and PP groups by 6 and 9 dph, respectively (*P* < 0.05).

From 9 dph, the steroid in the MT treatment continuously increased and reached a maximum of 481.29 ± 23.42 pg mL^− 1^ at 120 dph. The T concentration in the PP-treated fish also steadily increased from 30 dph and reached a significantly similar level as in the MT group from 45 to 90 dph (*P* > 0.05). However, at 120 dph, the same steroid became significantly higher in the MT-treated fish than in the ones exposed to PP (*P* < 0.001). In the CT treatment, the steroidal profile increased slowly from 6 dph and thus remained lower than in the MT-treated fish until 120 dph. The T level was similar between the PP and CT treatments from 3 to 21 dph, and thereafter became significantly higher in the PP-treated fish until the end of the experiment.

### Ratio of 11-ketotestosterone to 17β-estradiol among treatments

**Fig. 6:**
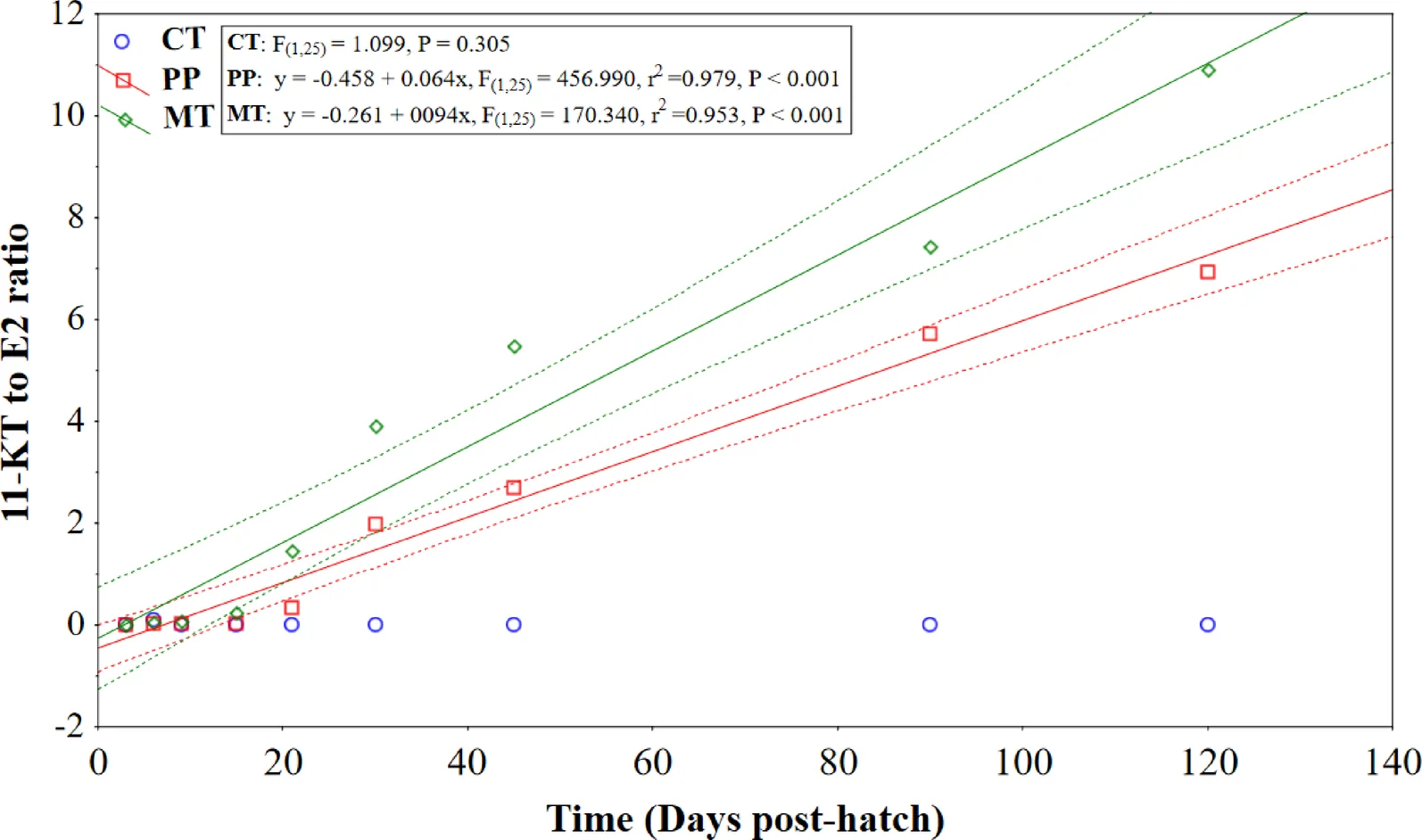
The change in the 11-ketotestosterone (11-KT) to 17β-estradiol (E2) ratio in the gonadal tissues of Nile tilapia over time. The fish were fed only on a basal diet (CT), or the same basal diet supplemented with 1,280 mg pine pollen kg^-1^ diet (PP), or the same basal diet supplemented with 60 mg 17α-methyltestosterone hormone kg^-1^ diet (MT). The dotted lines represent 95% confidence bands

An increasing trend of 11-KT to E2 ratio was observed in PP (y = − 0.458 + 0.064x, F _(1, 25)_ = 456.990, r^2^ =0.979, *P* < 0.001; Fig. 6) and MT (y = − 0.261 + 0094x, F _(1, 25)_ = 170.340, r^2^ = 0.953, *P* < 0.001; Fig. 6) treatments. No significant relationship between the ratio and time was found in the fish from the CT treatment (Regression analysis; F _(1, 25)_ = 1.099, *P* = 0.305; Fig. 6).

Among the treatments, the 11-KT to E2 ratio was influenced by a significant interaction between dietary treatment and time (RM-ANOVA; F _(16, 48)_ = 15.984, *P* < 0.001; Fig. 6). The ratio was higher in the PP and MT-treated fish than in the CT fish for the entire period of the experiment. By 15 dph, the ratio was significantly higher in the MT-treated fish than in those from the CT treatment and remained so till 120 dph (*P* < 0.05). Similar findings were observed in the PP-treated fish from 3 to 120 dph (*P* < 0.05). Notably, a significant rise in the 11-KT/E2 ratio was found in both MT and PP treatments between 21 and 30 dph, from 0.324 ± 0.161 to 1.972 ± 0.372, and 1.450 ± 0.494 to 3.901 ± 0.895, respectively (MT: *P* < 0.05). Meanwhile, the ratio was significantly higher in the MT treatment than in the PP group from 21 dph until the end of the experiment (*P* < 0.05).

### Expression of male-specific genes among treatments

**Fig. 7:**
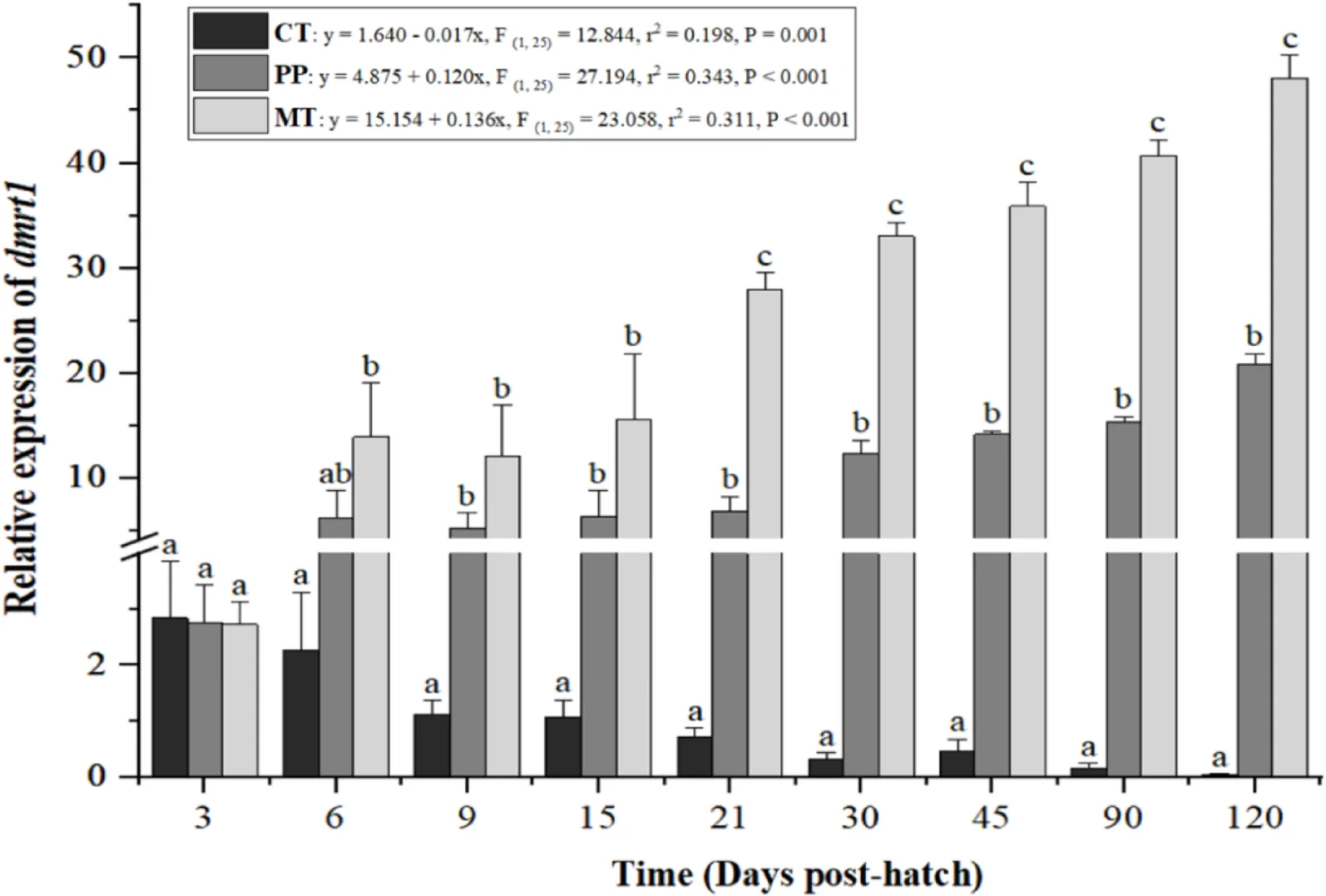
Mean (± standard error) relative expression of doublesex and mab-3 related transcription factor (*dmrt1*) in the trunks (3 to 21 dph) and gonads (30 to 120 dph) of Nile tilapia fed: (1) only basal diet (CT); (2) the same basal diet supplemented with 1,280 mg of PP kg^-1^ (PP); or (3) the same basal diet supplemented with only 60 mg of 17α-methyltestosterone hormone (MT) kg^-1^. Each bar represents the mean of the normalised gene expression ratio of six individuals on each sampling day. Different superscript letters above the error bars indicate significant differences in gene expression among treatments in the same time interval (Tukey’s HSD post-hoc, *P* < 0.05). The regression model equations describe the change in *dmrt1* expression over time for each treatment

**Fig. 8:**
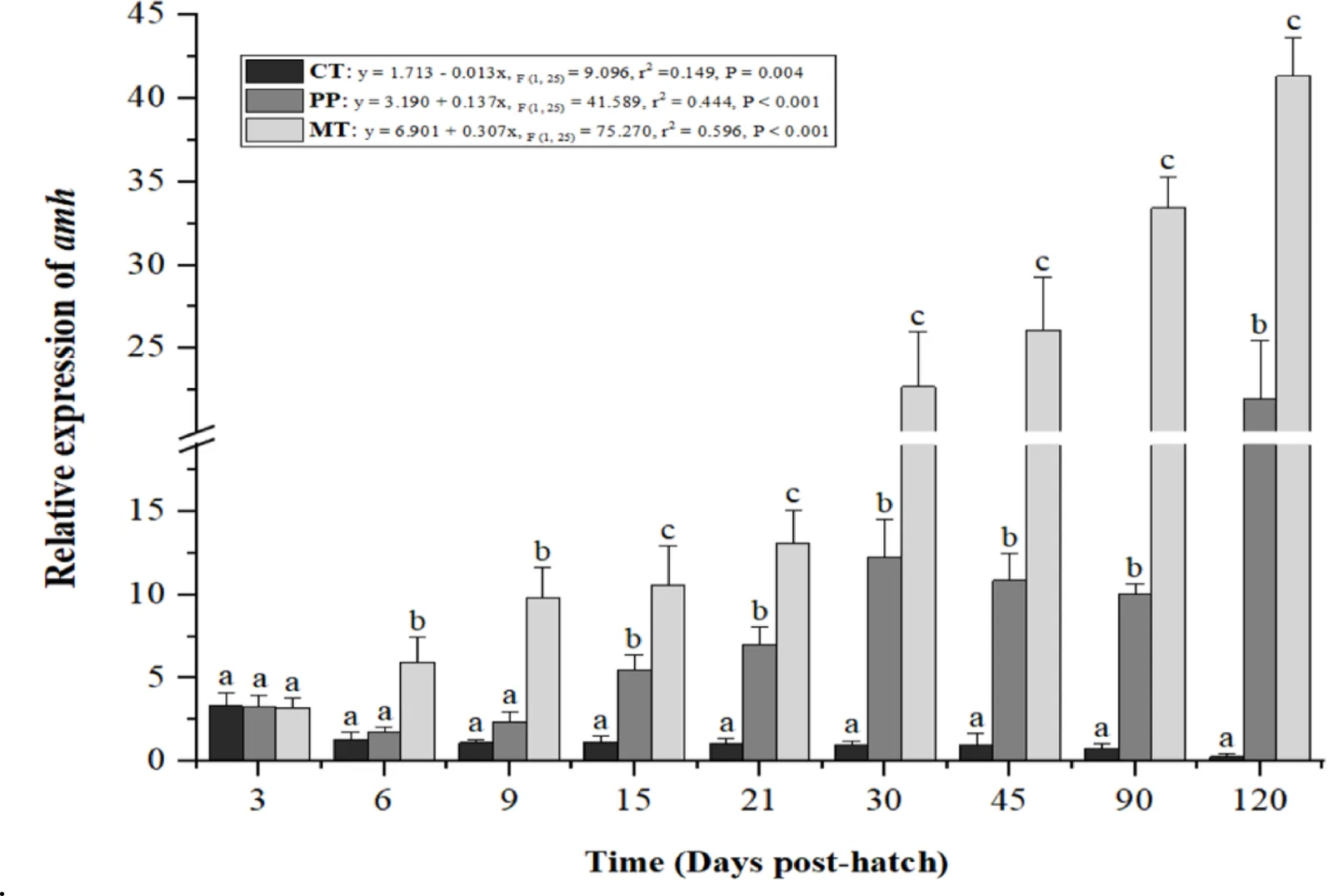
Mean (± standard error) relative expression of anti-müllerian hormone (*amh*) in the trunks (3 to 21 dph) and gonads (30 to 120 dph) of Nile tilapia. The fish were fed only a basal diet (CT), or the same basal diet supplemented with 1,280 mg of PP kg^-1^ (PP), or the same basal diet supplemented with only 60 mg of 17α-methyltestosterone hormone (MT) kg^-1^. Each bar represents the mean of six individuals’ normalised gene expression ratio on each sampling day. Different superscript letters above the error bars denote significant differences in gene expression among treatments in the same time interval (Tukey’s HSD post-hoc, *P* < 0.05). The regression equations describe the change in *amh* expression over time for each treatment

The expression of the *dmrt1* and *amh* was influenced significantly by the interaction between dietary treatment and time, with the expression changing differently over time among the treatments (RM-ANOVA; *dmrt1*: F _(16, 48)_ = 4.985, *P* < 0.001; Fig. 7 and *amh*: F _(16, 48)_ = 10.754, *P* < 0.001; Fig. 8). In the CT group, expression declined significantly from 3 to 120 dph (*dmrt1*: y = 1.640–0.017x, F _(1, 25)_ = 12.844, r^2^ = 0.198, *P* = 0.001; Fig. 7 and *amh*: y = 1.713–0.013x, F _(1, 25)_ = 9.096, r^2^ =0.149, *P* = 0.004; Fig. 8). Between 3 and 30 dph, *dmrt1* and *amh* were down-regulated by 8.3- and 3.4-fold, respectively, followed by further decreases of 5.8- and 3.5-fold between 30 and 120 dph. Conversely, both PP and MT treatments induced significant up-regulation of *dmrt1* and *amh*. In PP-treated fish, transcript abundance increased linearly over time (*dmrt1*: y = 4.875 + 0.120x, F _(1, 25)_ = 27.194, r^2^ = 0.343, *P* < 0.001; Fig. 7; *amh*: y = 3.190 + 0.137x, F _(1, 25)_ = 41.589, r^2^ = 0.444, *P* < 0.001; Fig. 8), while a stronger increase was observed in MT-treated fish (*dmrt1*: y = 15.154 + 0.136x, F _(1, 25)_ = 23.058, r^2^ = 0.311, *P* < 0.001; Fig. 7; amh: y = 6.901 + 0.307x, F _(1, 25)_ = 75.270, r^2^ = 0.596, *P* < 0.001; Fig. 8) treatments. From 3 to 30 dph, *dmrt1* expression increased by 12.1- and 3.4-fold in the MT and PP groups, respectively, whereas *amh* increased by 7.0- and 3.7-fold in the same treatments. Between 30 and 120 dph, expression continued to rise, but at a lower rate, with dmrt1 increasing by 1.5- and 1.9-fold, and amh by 1.8-fold, in both MT and PP groups.

Significant differences among treatments were evident from 6 dph onwards. For *dmrt1*, transcript levels were significantly elevated from 15 to 30 dph in MT-treated fish and from 21 to 30 dph in PP-treated fish (*P* < 0.05), with MT fish showing significantly higher expression than PP fish from 21 to 120 dph (*P* < 0.05). Similarly, *amh* expression in PP-treated fish exceeded that of CT fish from 15 to 120 dph (*P* < 0.05), whereas MT-treated fish displayed significantly higher expression than both CT and PP groups from 6 dph throughout the study (*P* < 0.05).

### Expression profiles of female-specific genes in the experimental fish

**Fig. 9:**
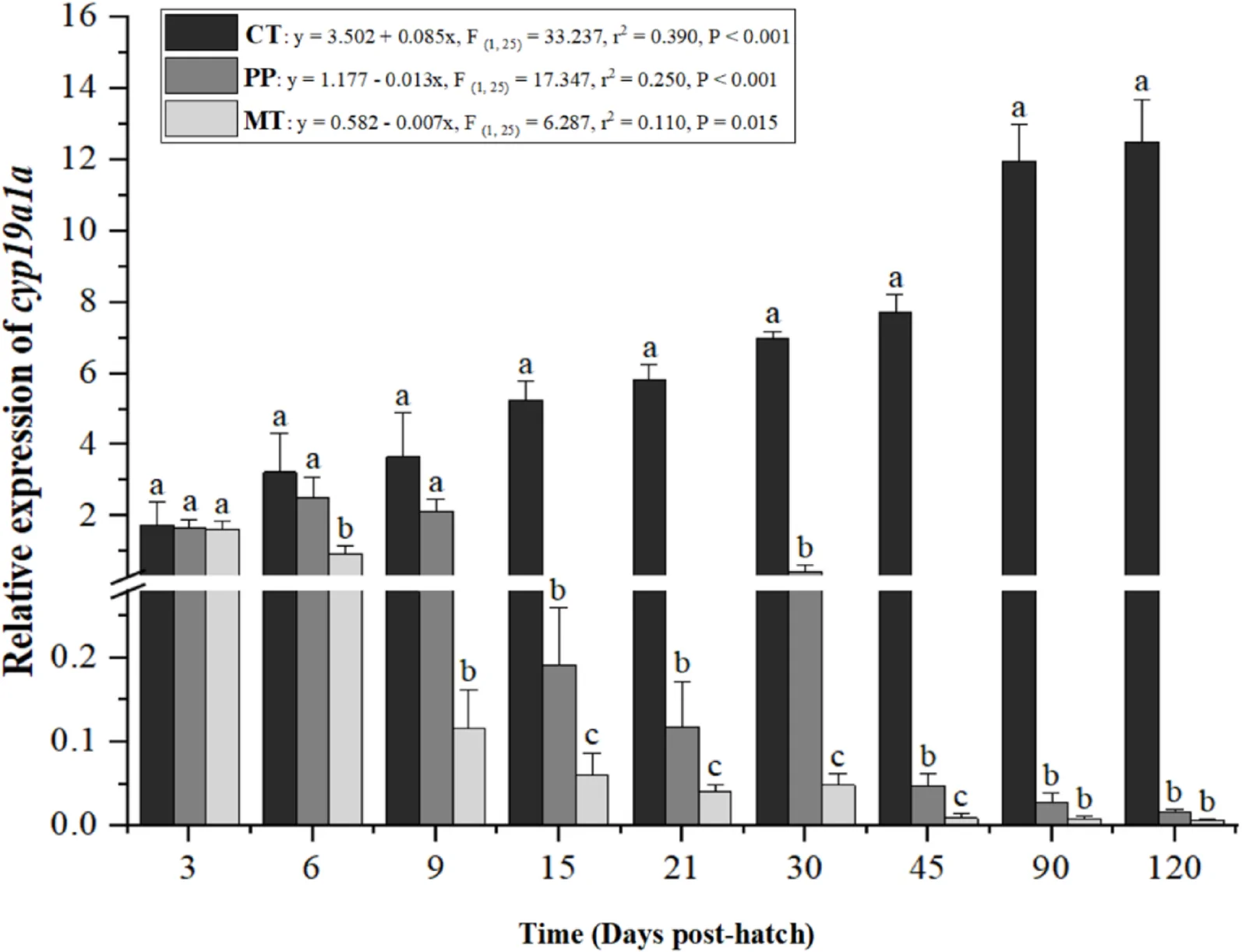
Mean (± standard error) relative expression of cytochrome P450, family 19, subfamily A, polypeptide 1a (*cyp19a1a*) in the trunks (3 to 21 dph) and gonads (30 to 120 dph) of Nile tilapia. Fish were fed only a basal diet (CT), or the same basal diet supplemented with 1,280 mg of PP kg^-1^ (PP), or a basal diet complemented with 60 mg of 17α-methyltestosterone hormone (MT) kg^-1^ diet. Each bar represents the mean of the normalised gene expression ratio of six individuals on each sampling day. The different superscript letters above the error bars indicate significant differences among treatments (*P* < 0.05) in the same time interval (Tukey’s HSD post-hoc, *P* < 0.05). The regression model equations describe the trend in the expression of *cyp19a1a* over time for each treatment

**Fig. 10:**
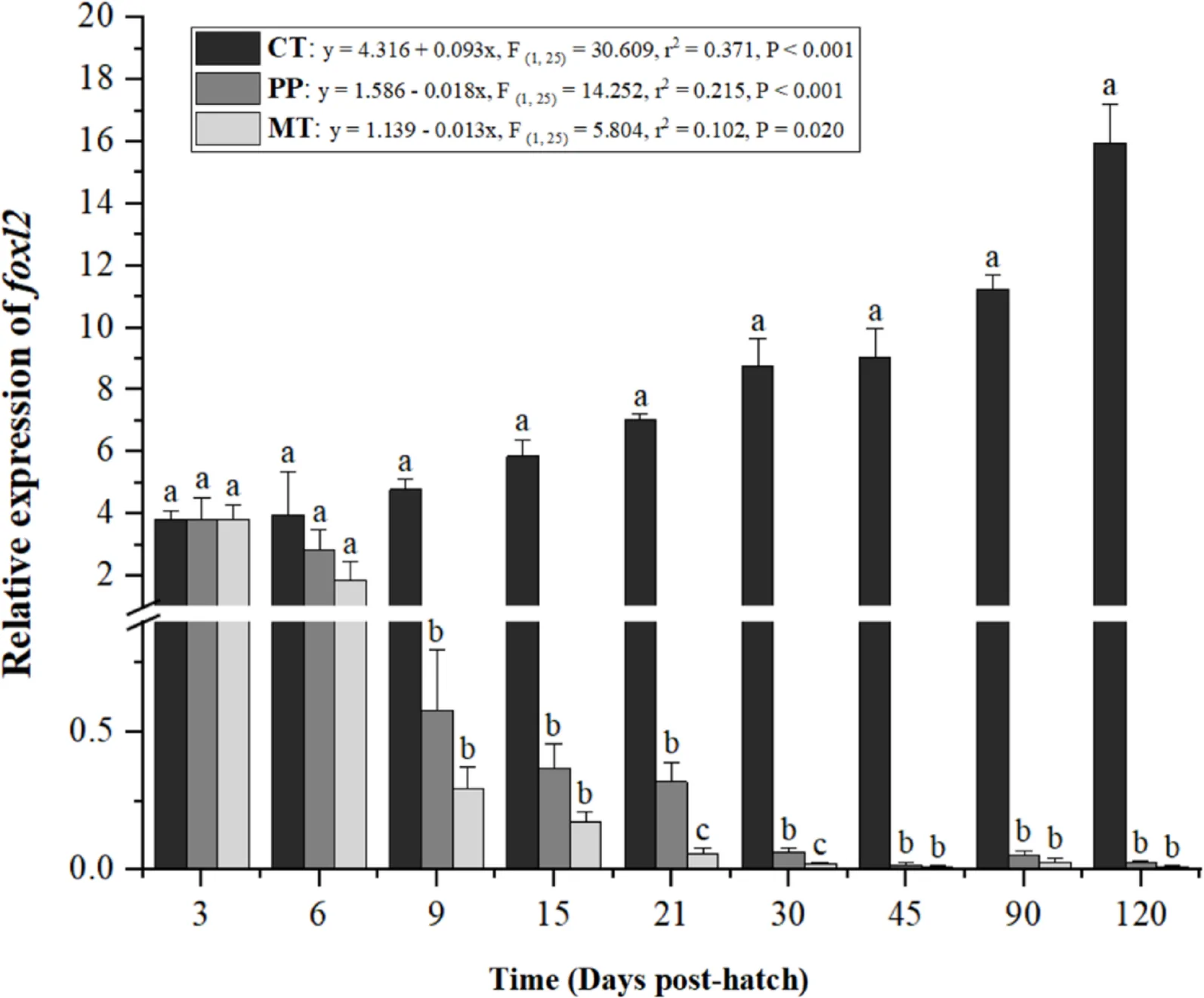
Mean (± standard error) relative expression of forkhead box protein L2 (*foxl2*) in the trunks (3 to 21 dph) and gonads (30 to 120 dph) of Nile tilapia. The fish were fed only a basal diet (CT), or the same basal diet supplemented with 1,280 mg of PP kg^-1^ (PP), or the same basal diet supplemented with only 60 mg of 17α-methyltestosterone hormone (MT) kg^-1^. Each bar represents the mean of six individuals’ normalised gene expression ratio on each sampling day. Different superscript letters above error bars indicate significant differences in the expression of the gene among treatments in the same time interval (Tukey’s HSD post-hoc, *P* < 0.05). The regression model equations indicate the trend in *foxl2* expression over time for each treatment

The expression of the female-specific genes *cyp19a1a* and *foxl2* was significantly affected by the interaction between dietary treatment and time (RM-ANOVA; *cyp19a1a*: F _(16, 48)_ = 4.330, *P* < 0.001; Fig. 9 and *foxl2*: F _(16, 48)_ = 8.377, *P* < 0.001; Fig. 10). Both genes displayed expression patterns opposite to those observed for the male transcripts. In the CT group, transcript abundance increased linearly from 3 to 120 dph (*cyp19a1a*: y = 3.502 + 0.085x, F _(1, 25)_ = 33.237, r^2^ = 0.390, *P* < 0.001; Figs. 9 and *foxl2*: y = 4.316 + 0.093x, F _(1, 25)_ = 30.609, r^2^ = 0.371, *P* < 0.001; Fig. 10). From 3 to 30 dph, *cyp19a1a* increased by 4.0-fold and *foxl2* by 2.3-fold, followed by additional increases of 1.8-fold for both genes between 30 and 120 dph. In contrast, PP and MT treatments resulted in sustained down-regulation of both genes. In PP-treated fish, expression declined significantly over time (*cyp19a1a*: y = 1.177–0.013x, F _(1, 25)_ = 17.347, r^2^ = 0.250, *P* < 0.001; Fig. 9 and *foxl2*: y = 1.586–0.018x, F _(1, 25)_ = 14.252, r^2^ = 0.215, *P* < 0.001; Fig. 10), while a similar but generally stronger decline occurred in MT-treated fish (*cyp19a1a*: y = 0.582–0.007x, F _(1, 25)_ = 6.287, r^2^ = 0.110, *P* < 0.001; Fig. 9 and *foxl2*: y = 1.139–0.013x, F _(1, 25)_ = 5.804, r^2^ = 0.102, *P* = 0.020; Fig. 10). Between 3 and 30 dph, *cyp19a1a* decreased by 33.5-fold in MT-treated fish and by 3.7-fold in PP-treated fish, whereas *foxl2* was down-regulated by 165.3- and 59.1-fold, respectively. From 30 to 120 dph, *cyp19a1a* decreased further by 25.6-fold in PP-treated fish and by 6.7-fold in MT-treated fish, while *foxl2* declined by 2.4-fold in both treatments.

No significant differences in *cyp19a1a* expression were observed between CT and PP fish from 3 to 9 dph. However, expression was significantly higher in CT fish from 15 to 120 dph and remained significantly lower in MT fish than in CT fish from 6 to 120 dph (*P* < 0.05). Furthermore, *cyp19a1a* expression was significantly lower in MT fish than in PP fish from 6 to 45 dph, although no differences were observed at 90 and 120 dph. A similar pattern was observed for *foxl2*, whose expression remained significantly lower in PP- and MT-treated fish than in CT fish from 9 to 120 dph (*P* < 0.05). The MT treatment caused significantly stronger suppression than the PP treatment at 21 and 30 dph (*P* < 0.05), whereas expression levels did not differ between treatments from 45 to 120 dph.

However, similar expression levels were observed at 90 and 120 dph in both the PP and MT groups. For *foxl2*, expression level in the CT treatment was up-regulated throughout the experiment. However, the gene was down-regulated in the PP- and MT-treated fish and remained significantly lower than in the CT group from 9 to 120 dph (*P* < 0.05). Notably, *foxl2* was significantly attenuated in the MT-treated fish than in the fish from the PP treatment from 21 to 30 dph (*P* < 0.05). However, from 45 to 120 dph, the gene was down-regulated in a similar manner by both PP and MT treatments.

## Discussion

Bioactive compounds from plant extracts, especially saponins, flavonoids, alkaloids, and steroids, are known to induce female-to-male sex change in fish [62, 98–100]. Evidently, phyto-extracts from pine pollen (PP) induced masculinisation in Nile tilapia [70, 85–87]. However, the mode of action of the PP and other plant extract to direct sex differentiation in fish remained unknown. The present study describes the mechanism underlying pine pollen (PP)-induced sex inversion in Nile tilapia. Specifically, the study examined the temporal expression patterns of sex-specific genes, steroid profiles, and histological changes in the trunk tissues (3–21 dph) and gonads (30–120 dph) of PP-treated all-female Nile tilapia. Notably, trunk tissues were used from 3 to 21 dph because the fish were too small for reliable gonad excision, which represents a limitation as gene expression and steroid measurements may include contributions from non-gonadal tissues.

The experimental fish were treated with PP and MT from 3dph onwards, as the gonads of Nile tilapia at this stage are still undifferentiated with potential to develop into either an ovary or a testis [3, 101]. Fish were exposed to the treatments from the onset of first feeding (3 dph) to 30 dph, to match the sensitive period for gonadal differentiation [3, 102, 103]. Gonadal differentiation involved the proliferation of testicular germ cells in PP and MT-treated fish, while oogonia and oocytes increased in the CT group [104–106]. Between 3 and 15 dph, the germ cells were generally morphologically similar in all treatments, with no distinct testicular or ovarian structures visible. During this period, the primordial gonad of the fish gradually loses its intersexual characteristics, and the phenotypic sex becomes fixed [3, 31, 107]. By 15 dph, MT treated fish had significantly repressed female germ cell proliferation, with a similar pattern observed in PP-treated fish by 21 dph. This early suppression of the female germ cell development was followed by testicular differentiation in PP and MT-treated fish between 21 and 45 dph, coinciding with the critical period for male sex differentiation in Nile tilapia [3, 107–109]. In contrast, normal ovarian development in the CT group was evident between 21 and 30 dph, as ovarian differentiation occurs earlier than testicular differentiation [109]. Although distinct gonadal differentiation was observed histologically, it is recommended that molecular markers be used to confirm the correspondence between genetic and phenotypic sex [9]. The use of molecular markers in future studies would improve the accuracy of sex determination and provide robust validation of PP and MT treatment effects.

Consistent with the gonadal histological observations, the period of female-to-male sex change in the PP and MT treatments was marked by down-regulation of *cyp19a1a*, *foxl2*, and E2, accompanied by up-regulation of *dmrt1*, *amh*, and 11-KT. In contrast, increased expression of *cyp19a1a*, *foxl2*, and E2 was observed in the CT group and was associated with female phenotype differentiation. Given that all-female fish were used in the study, higher E2 levels were observed at 3 days post-hatch (dph), reflecting yolk-derived maternal estrogens rather than de novo synthesis by the embryo or larval gonad system [1, 73]. Subsequently, E2 levels showed treatment-specific responses, with a decline in PP- and MT-treated fish and an increase in CT-treated fish. These findings suggest that androgenic compounds in PP and MT were key in repressing female sex genes and steroids while promoting male-related genes and steroids during sex differentiation, consistent with previous studies [48, 110–115]. Additionally, E2 suppression in the experimental fish triggered the differentiation of Leydig cells, male-specific secondary sex characteristics, and sexual behaviour [65]. Notably, a high 11-KT to E2 ratio was maintained in the PP and MT treatments from 6 dph onward, with a pronounced spike from 21 dph onward, coinciding with the onset of distinct testicular differentiation among the treatments. The steroid ratio controls sex differentiation, with excess steroid determining the direction of gonadal sex development regardless of the genetically determined sex. High concentrations of 11-KT favour the development of male sex phenotype, while elevated E2 promotes female sex differentiation [35, 52, 116, 117]. The notable differences in the 11-KT to E2 ratio among treatments suggest that this ratio plays a key role in controlling sex differentiation in Nile tilapia [31, 40, 41, 118–120]. The more advanced gonad development in fish from the MT treatment compared to the PP treatment is attributed to the higher 11-KT to E2 ratio.

The androgen-to-estrogen balance is regulated by the bio-conversion rates of T to either 11-KT or E2, which is controlled by the prevailing sex gene network [31, 40, 41, 118–120]. During the early stages of sex differentiation in Nile tilapia, the male- and female-specific genes act antagonistically, and the prevailing network controls steroid production, ultimately directing the development of an appropriate sex phenotype [3, 6, 24, 27, 51, 64, 106, 107, 121]. In the steroid synthesis cascade, *cyp19a1a* plays a key role in maintaining the sex steroid balance by converting androgens into estrogens, i.e., T to E2 [25, 31, 36, 122]. Notably, the expression of *cyp19a1a* is regulated by the antagonism between *foxl2*, together with *dmrt1* and *amh*. The *dmrt1* interacts antagonistically with *foxl2* to suppress the expression of *cyp19a1a*, which, in turn, inhibits estrogen production, with the hydroxylation of T to produce 11-KT, thereby activating male sex development [18, 19, 23, 26–29, 34, 123]. In contrast, *foxl2* activates *cyp19a1a* by binding to the gene promoter region, as well as interacting with steroidogenic factor 1 (*sf1*), triggering the conversion of T to E2 and, consequently, ovarian differentiation [19, 31, 34, 109, 124–127]. Therefore, a shift in the expression of these sex-biased genes alters the sex steroid profiles and, subsequently, the sex differentiation process [18, 42–44]. In the present study, the observed female-male gonadal transformation in PP and MT treatment could be attributed to up-regulation of *dmrt1* and *amh* in the fish, coupled with the suppression of *cyp19a1a* and *foxl2*, leading to decreased E2 levels and increased 11-KT profiles. Similarly, ovarian differentiation in CT-treated fish may have been driven by elevated expression of *cyp19a1a* and *foxl2*, along with suppression of *dmrt1* and *amh*, resulting in low 11-KT and increased E2 concentrations. These findings are consistent with previous studies showing that exogenous hormonal treatment alters the sex gene network and steroid synthesis, particularly the 11-KT to E2 balance, thereby directing sex differentiation in Nile tilapia [6, 18, 19, 24, 30, 31, 33, 47, 49, 50, 54, 65, 128].

During the early stages of sex differentiation, expression of the sex-biased genes was low, but it subsequently increased or decreased over the course of the experiment, depending on the treatment. Related to this, the steroid levels followed a comparable pattern, which could be due to the prevailing sex direction. The results imply that, in addition to directing the gonadal differentiation process, the genes and steroids under study were also crucial for maintaining the gonadal phenotype [3, 5, 106]. Notably, prior to gonadal differentiation, a high-fold increase in male-specific gene expression and 11-KT levels was observed in the PP and MT treatments, whereas female-related genes and E2 levels were elevated in the CT group. This pattern coincided with the sensitive period for sex differentiation, during which masculinisation occurs in Nile tilapia [48, 110–115]. Besides, the expression trend underscores the roles of *dmrt1*, *amh*, and 11-KT in promoting male sex differentiation and of *cyp19a1a*, *foxl2*, and E2 in female sex development in Nile tilapia [3, 14, 19, 20, 27, 31, 34, 63, 106, 129–131]. Moreover, the male phenotype predominated in the PP and MT treatments, whereas only females were present in the CT group.

Overall, PP and MT followed a similar regulatory pattern for sex-biased genes and steroids, suggesting a related mechanism of sex inversion in the present study. The presence of steroids, mainly T, in PP could be responsible for the female-to-male sex change [62]. Like other androgens, PP suppressed the expression of *cyp19a1a*, thereby inhibiting the conversion of T to E2 and triggering female-to-male sex change [9, 27, 46, 52, 132–136]. The higher masculinisation potency of MT compared with PP could be attributed to the stable nature of MT, which minimises aromatisation and hence more hormone is bioavailable to the fish, inducing higher expression of *dmrt1* and *amh* in the MT-treated fish [137, 138]. The PP treatment was less effective than MT at inducing masculinization, possibly because of the low steroid quantities in PP. Since the concentration of active ingredients in pollen varies with season, geographical origin, and environmental factors [139], the present study recommends characterizing pollen from diverse sources to identify those with high steroid content, while accounting for these variables. Also, optimising the processing method, storage duration, and conditions is crucial to prevent or minimise the loss of target bioactive compounds [140, 141].

Although sex change is more responsive to exogenous hormonal treatment in sexually undifferentiated fish, reports indicate a second window for sex reversal, where already differentiated individuals undergo sex change. An antagonistic effect continues to exist between the male and female sex networks after gonadal differentiation, hence allowing for sexual plasticity [33, 47, 64, 66, 142]. The second window for sex change, designated as secondary sex reversal [48, 111, 112] could be harnessed to achieve better masculinisation results when using PP. In the present study, some female individuals were still present after exposure of all-female Nile tilapia progeny to PP between 3 and 30 dph, suggesting that extending the treatment period may be necessary. Earlier studies that utilised PP for sex inversion in Nile tilapia [85] and African catfish (*Clarias gariepinus*) [143] reported higher proportions of male individuals, after feeding the fish PP-supplemented feeds for 56 and 72 days, respectively. It is vital that during the extended exposure period of Nile tilapia to PP, genetic XY progeny are included to provide more information on the extent of up-regulation of male sex genes in the sex-inversed XX female fish.

## Conclusion

This is the first description of the mechanism underlying PP-induced sex inversion of Nile tilapia. Pine pollen altered the expression of sex-biased genes and steroid profiles, disrupting the molecular and endocrine sex differentiation pathways that ultimately determine the phenotypic sex of Nile tilapia.

## Data Availability

The data that support the findings of this study are accessible from the corresponding author upon request.
